# Choline and N-acetyl aspartate levels in the dorsolateral prefrontal cortex at the beginning of the recovery phase as markers of increased risk for depressive episode recurrence under different duration of maintenance therapy and after it: a retrospective cohort study

**DOI:** 10.3325/cmj.2018.59.244

**Published:** 2018-10

**Authors:** Neven Henigsberg, Aleksandar Savić, Marko Radoš, Helena Šarac, Milan Radoš, David Ozretić, Maja Bajs Janović, Viktorija Erdeljić Turk, Ana Šečić, Petra Kalember, Pero Hrabač

**Affiliations:** 1 Croatian Institute for Brain Research, University of Zagreb School of Medicine, Zagreb, Croatia; 2 Department of Psychiatric Research, University Psychiatric Hospital Vrapče, Zagreb, Croatia; 3 Department of Diagnostics and Intensive Care, University of Zagreb School of Medicine, Zagreb, Croatia; 4 Department of Clinical Pharmacology, University Hospital Center Zagreb, Zagreb, Croatia; 5 Clinic for Rheumatology, Physical Medicine and Rehabilitation, University Hospital Center *Sestre Milosrdnice*, Zagreb, Croatia; 6 Polyclinic Neuron, Croatian Institute for Brain Research, University of Zagreb School of Medicine, Zagreb, Croatia; * NH and AS contributed equally

## Abstract

**Aim:**

To evaluate the relationship between the dynamics of proton magnetic resonance spectroscopy (1H-MRS) brain metabolite levels at the beginning of the recovery phase of the index depressive episode and the time to the recurrence of depression.

**Methods:**

This retrospective cohort study analyzed the changes in N-acetyl aspartate (NAA), choline (Cho), and glutamate-glutamine in 48 patients with recurrent depression treated with maintenance antidepressant monotherapy at a stable dose. 1H-MRS was performed at the start of the recovery phase and 6 months later. 1H-MRS parameters, index episode descriptors, and depressive disorder course were analyzed by Cox proportional hazards model.

**Results:**

NAA and Cho decrease six months after the beginning of the recovery period were time-independent risk factors for depressive episode recurrence. Hazard ratio associated with NAA decrease was 2.02 (95% confidence interval 1.06-3.84) and that associated with Cho decrease was 2.06 (95% confidence interval 1.02-4.17). These changes were not related to symptoms severity, as Montgomery-Asberg Depression Scale score remained generally unchanged (mean -0.01; standard deviation 1.6) over the first 6 months of recovery.

**Conclusion:**

Patients receiving maintenance antidepressant therapy after recovery who experience a decrease in NAA or Cho levels early in the recovery phase have a double risk of depressive episode recurrence. Sustained NAA and Cho levels at the beginning of the recovery phase may indicate increased brain resilience conferred by antidepressant therapy, while NAA and Cho decrease may indicate only the trait-related temporal effect of therapy in another stratum of patients.

With lifetime prevalence of up to 15%, the burden of depression in the form of mortality (suicide, cardio- and cerebrovascular risk), loss of quality of life, and psychosocial and work disability, amounts to significant impairment on multiple levels of functioning (1). We now accept the Kraepelinian view of affective disorder as having a remitting and recurring course, with some residual symptoms persisting in a number of patients even after entering remission (eg, cognitive symptoms) (1,2). Although there are different ways of conceptualizing and defining remission, recovery, relapse, and recurrence, we generally define remission by a significant reduction in symptoms lasting for a specific time period (3 consecutive weeks). It is largely considered that recovery is achieved after at least 4 months of remission without a relapse (3-5). Depression recurrence is defined as a new depression episode after the onset of recovery phase, although some authors question any meaningful difference between relapse and recurrence (3,5).

A study of 175 outpatients treated with antidepressants for 6 months, and monitored for one year, showed that 46% of those who were not depressed at 6 months had another episode of depression (6). Another study indicated that the recurrence rate after 15 years in primary care population was around 35%, while in specialized mental health settings it rose to as high as 85% (7). The risk of repeated depressive episode decreases over time spent in recovery without recurrence, but it also becomes more pronounced after each subsequent episode, rising to above 90% after 3 episodes and approaching 100% without prophylactic treatment (8,9). Most important predictors of repeated depressive episode are residual symptoms and the number of previous episodes, but also early onset, family history of recurrent depression, history of dysthymic disorder, severity of the first or index episode, as well as negative cognitive patterns, high neuroticism, poor social support, and stressful life events (8,10,11).

Research consistently confirms significant reduction in the odds of relapse with antidepressants treatment, but given the long-term nature of depressive disorders and the risk of recurrence there is an ongoing debate on the recommended treatment duration (12,13). Most guidelines recommend continued treatment for 4-6 months after a depression episode, and there is evidence for the benefit of longer maintenance treatment (13,14).

The dorsolateral prefrontal cortex (DLPFC) is considered as a region of interest in depression. Changes in its activity relative to the phase of illness and treatment, and strong indications of neurodegenerative processes in the DLPFC as a result of relapse and illness chronicity are well documented (15-17). Proton magnetic resonance spectroscopy (1H-MRS) allows for the evaluation of changes in brain metabolites in vivo*,* and can improve our understanding of neuronal changes in the brain on molecular and submolecular levels. 1H-MRS can identify various neurochemicals, such as N-acetyl aspartate (NAA) and choline-containing metabolites (Cho). They are usually reported as a ratio to creatine (Cr), as its brain levels are considered to be relatively stable. Glutamate, glutamine, and gamma aminobutyric acid (GABA) metabolites are commonly combined in glutamate-glutamine (Glx) peak due to their overlap. NAA with its presence in neurons is considered to be a putative marker of neuronal integrity and functionality (18-20). Previous research has shown NAA changes following various modalities of antidepressant treatment (21,22). Cho is considered to be a potential marker of cellular membrane turnover, and previous research reported elevated Cho/Cr ratio in ventral prefrontal white matter bilaterally after 12 weeks of treatment with selective serotonin reuptake inhibitors (SSRIs) (23). Reduced Glx values in the prefrontal cortex of depressed patients correlate with disease severity, and GABA levels normalize following antidepressant treatment (24-26). Further longitudinal research on the DLPFC and other frontal regions is warranted in order to determine functional relevance of metabolite changes in depression treatment (27).

Our previous 1H-MRS study on the same cohort of patients evaluated the risk of depressive symptoms recurrence in patients on a stable dose of antidepressant in maintenance therapy. Patients who experienced recurrence during maintenance treatment were compared with those who entered the discontinuation of antidepressants phase (28). Decreased Cho/Cr and NAA/Cr ratios after recovery were related to an elevated risk for recurrence during maintenance therapy, and decreased Cho/Cr was associated with a 2.7-fold increase in the risk of depression recurrence (28).

The main shortcoming of our past research was that the end-point of the study was defined as either start of antidepressant withdrawal or recurrence of a depressive episode, whatever occurs earlier. The present study aims to provide more detailed insight into correlates between neurochemical changes and clinical outcomes of initiated maintenance therapy, without imputing future knowledge on treatment resolution to a historical time point, so to avoid a “recurrence agnostic” approach. For this purpose, we collected data on eventual recurrent episodes for patients who survived episode-free to medication withdrawal point and analyzed survival data for all recruited patients. We hypothesize that restructuring of the DLPFC continued in the recovery period and would reflect in an increase in NAA and Cho levels at the recovery, both markers contributing to longer episode-free survival period.

## Participants and methods

**Figure 1:**
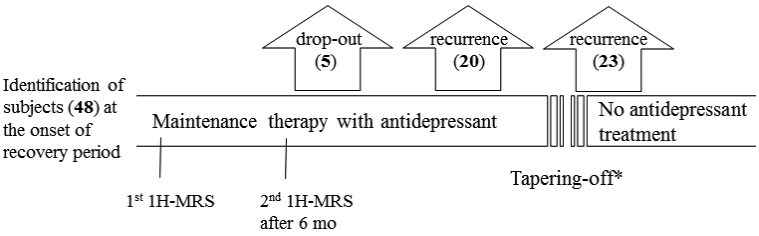
Study course and recurrence with respect to the phase during which it took place. *Duration of maintenance antidepressant therapy, tapering-off, and discontinuation were determined by attending clinicians and vary between cases. Figure illustrates only relative temporal relationship between scans, maintenance antidepressant therapy, its discontinuation, and recurrences. The distance between the elements in the illustration is not to be interpreted as a scaled-down version of real temporal relationships. 1H-MRS – proton magnetic resonance spectroscopy.

This retrospective cohort study was performed in patients with recurrent depression who participated in past or ongoing research on identification of predictors of therapeutic response (28). The patients were diagnosed according to International Classification of Diseases (ICD)-10, and the diagnosis was confirmed by Mini International Neuropsychiatric Interview (MINI) 5.0 (29). We identified 48 patients who had (a) MRS scans of the DLPFC at the start of the recovery period, (b) a follow-up MRS scan 6 months later, and (c) who were on maintenance antidepressant therapy since entering the recovery phase (Figure 1).

Definition of recovery was based on the standard criterion of a Montgomery-Asberg Depression Scale (MADRS) score ≤10 (30), and MRS scan was performed on the day of clinical evaluation (or at the latest 2 days following clinical evaluation). The first 1H-MRS evaluations were done within 4 months of remittance of the depressive symptoms (recovery phase) and the second evaluations were done 6 months after. Patients continued with prescribed maintenance therapy either until withdrawal (beginning of tapering off of antidepressant) or until the recurrence of the depressive episode. There were no specific study requirements regarding the choice of antidepressant medication, conditions for the antidepressant change, or the antidepressant treatment duration, and these decisions were made by the three attending psychiatrists at the Neuron Polyclinic. Patients were assessed on outpatient basis by psychiatric interviews and MADRS at least every 6 months and followed up until their next depressive episode. Monotherapy with antidepressants was used (following Summary of Product Characteristics for each of the individual antidepressants), and for some patients a stable dose of medication from benzodiazepine class, but no other additional therapeutic interventions (eg, psychotherapy), was used. Study protocol was formulated in accordance with the Declaration of Helsinki, and was approved by the Ethics Committee of the University of Zagreb School of Medicine (No. 04-1423-2006 of February 17, 2006) and the Ethics Committee of Polyclinic Neuron (No. 17-06 of March 23, 2006). All included patients previously signed informed consent for the participation in long-term evaluations. First person first visit and last person last visit were in April 2006 and July 2008, respectively.

1H-MRS was performed by two trained radiologists (MaR and MiR), blinded to the participants’ diagnosis using a 2.0 T system (Gyrex 2T-Prestige, GEMS/Elscint, Haifa, Israel) with a quadrate head coil. Head motion was minimized using foam pads, and the intersection of the frontal bone and two nasal bones (nasion) was used as a landmark for the scan. The voxels were repositioned in the area of interest – the left DLPFC, and spectroscopic volume of interest (15 × 15 × 15 mm) was selected in that region in order to minimize the amount of cerebrospinal fluid contained in the volume of interest. A point-resolved spectroscopy sequence was used (1500/35 [TR/TE – repetition time/echo time), with 100 averages. The spectra were reevaluated for peak NAA (at 2.02 ppm), Cho (at 3.2 ppm), Glx (at 2.2-2.4 ppm), and Cr (at 3.03 ppm). We used absolute NAA, Cho, and Glx values, as well as their ratios to Cr. The software package program supplied by the manufacturer (Gyrex 2T-Prestige, GEMS/Elscint) was used in the analysis of spectral data set.

### Statistical analysis

Candidate markers of prognosis entered into the analysis included brain metabolites in the region of interest (DLPFC), factors previously identified as important based on our current knowledge regarding recurrence of depressive episodes, and a set of factors that might modify the levels of analyzed brain metabolites. The model entered into Cox proportional hazards (CPH) analysis initially consisted of three sets of variables: 1) changes in NAA, Cho, and Glx expressed as ratio to Cr; 2) three variables describing the course of the last/index episode (time to remission, MADRS score improvement till recovery, and change in the MADRS score between the start of the recovery and 6-months follow-up); and 3) three variables describing the previous course of recurrent depressive disorder (age of the onset of the first episode, years lived with depression, and number of prior episodes). Forward stepwise likelihood ratio model was used. We checked proportional hazard assumption by constructing a product between the variable and a linear function of time, added an interaction term, and tested for its significance. After significant parameters were identified in the initial model, a model with dichotomized variables was developed (using overall direction of change and not the level of increase/decrease) in order to ensure easier identification and interpretation of changes in brain metabolites. The level of statistical significance was set at 0.05. Statistical analysis was performed in Statistica software package, version 13.2 (Dell Inc., Round Rock, TX, USA, licensed to the University of Zagreb School of Medicine).

## Results

The study included 30 female and 18 male patients at the start of their recovery phase. Recurrent episode was documented in 43 patients. In 20 patients, a new depression episode occurred while on maintenance therapy, and in 23 the initiation of antidepressant tapering off took place before the occurrence of another episode. With regards to 5 remaining patients, 2 were lost to follow-up, 2 converted to bipolar affective disorder, and 1 experienced serotonergic syndrome and was taken off of antidepressant.

The mean survival after the beginning of the recovery period (time of initiation of maintenance therapy) was 3.2 years (standard deviation [SD] = 2.0; Table 1). In 23 patients who went through antidepressant tapering off, recurrent episode appeared on average 1.4 years after its start (SD = 1.0).

**Table 1:** Descriptors of the disorder course and index episode

Characteristic	N	Mean (standard deviation)	Range
Disorder course descriptors			
age at onset of disorder	48	28.41 (8.57)	17-46
years lived with depression	48	15.4 (10.5)	2.0-40.8
No. of prior episodes	48	3.8 (2.0)	2-12
Current/index episode descriptors			
months to remission	48	5.7 (1.5)	2.7-8.9
Montgomery-Asberg Depression Scale score			
at the beginning of an episode	48	25.6 (4.1)	18-34
at the start of the recovery phase	48	5.4 (1.2)	3-9
at 6 months after the start of the recovery phase	48	5.4 (1.1)	2-8
at the last evaluation (recurrence)	43*	20.9 (2.3)	18-29

*2 patients were lost to follow-up, 2 converted to bipolar affective disorder, and 1 experienced serotonergic syndrome and was taken off of antidepressant.

Escitalopram was the most frequently used antidepressant (in 13 patients), followed by sertraline (n = 10), fluoxetine (n = 5), venlafaxine (n = 4), mirtazapine (n = 3), paroxetine (n = 3), citalopram (n = 2), amitriptyline (n = 2), reboxetine (n = 2), fluvoxamine (n = 2), moclobemide (n = 1), and imipramine (n = 1). According to Neuroscience-based Nomenclature-2 classification, the vast majority of antidepressants were reuptake inhibitors: serotonin transporter (n = 35); serotonin and norepinephrine transporters (n = 7); and norepinephrine transporter (n = 2). Three patients were treated with receptor antagonist (NE alpha-2, 5-HT2, 5-HT3) and 1 with reversible enzyme inhibitor.

Overall MADRS score significantly improved from the beginning of an episode to the start of the recovery phase, on average by 20.3 points (SD = 3.9.0), and did not change significantly in the following 6 months (-0.07; SD = 1.6).

**Figure 2:**
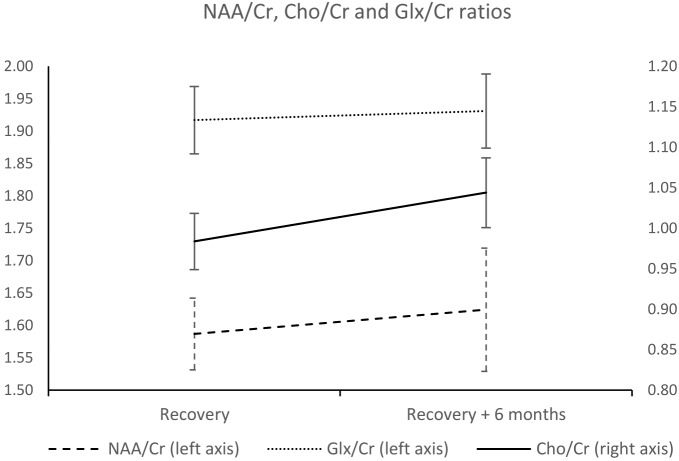
Magnetic resonance spectroscopy measurements of N-acetyl aspartate/creatine (NAA/Cr), choline-containing metabolites/creatine (Cho/Cr), and glutamate-glutamine/creatine ratios (means and 95% confidence intervals).

In the overall sample (Figure 2), Cho/Cr ratio increased significantly after 6 months (0.06; 95% confidence interval [CI] 0.028 to 0.092; *P* < 0.001), NAA/Cr increased moderately and non-significantly (0.037; 95% CI -0.031-0.106 to 0.106; *P* = 0.279), and Glx/Cr levels were nearly stable (0.014; 95% CI -0.055 to 0.083; *P* = 0.686). Only 14 out of 48 patients (29.2%) experienced a decrease in Cho/Cr, whereas nearly half of the patients experienced a decrease in NAA/Cr (23 patients, 47.9%). Changes in these two metabolites were not directly related to each other, as NAA/Cr decreased concurrently only in about half (8 of 14) of the patients who experienced Cho/Cr decrease. Changes in brain metabolites were not related to symptoms severity.

In CPH analysis, none of the time-dependent covariates was significant (*P* values of these covariates for NAA/Cr, Cho/Cr and Glx/Cr were 0.089, 0.218 and 0.721, respectively). This confirmed that there was no significant time-varying effect. In the assessment of proportionality assumption, constant hazard ratios were also confirmed visually by inspecting plot of the log(-log(S(t)) functions (Table 2).

**Table 2:** Parameter estimates of the initial model

Factor	Relative risk (95% confidence interval)	*P*
1H-MRS metabolite changes 6 months after recovery		
N-acetyl aspartate/creatine	0.084 (0.015-0.480)	0.005
choline/creatine	0.013 (0.001-0.328)	0.008
glutamate-glutamine/creatine	0.302 (0.062-1.459)	0.136
Current episode descriptors		
months to remission	0.847 (0.639-1.121)	0.245
MADRS improvement at recovery	1.065 (0.972-1.167)	0.178
MADRS change 6 months after recovery	1.075 (0.857-1.350)	0.531
Disorder course descriptors		
age at onset	0.993 (0.947-1.042)	0.776
years lived with depression	0.973 (0.934-1.014)	0.195
No. of prior episodes	1.101 (0.899-1.349)	0.351

*1H-MRS – proton magnetic resonance imaging; MADRS – Montgomery-Asberg Depression Scale.

Only two variables out of 9 entered into the initial CPH model had a significant contribution based on the inspection of beta coefficients, risk ratios with their 95% CI, t-value approximations, and Wald statistics. The two variables with significant contributions were the change in NAA/Cr and Cho/Cr, whereas all others had considerably lower contribution. This was further confirmed when all 9 variables were entered into CPH forward stepwise likelihood ratio model with two steps. After the inclusion of Cho/Cr change in the first step of the model (B = -3.762; SE = 1.495; Wald = 6.330; df = 1; *P* = 0.012; Exp(B) = 0.023), the final model (-2 log likelihood = 239.936; χ^2^ = 10.869; df = 2; *P* = 0.04) consisted of two variables: Cho/Cr change (B = -4.098; SE = 1.545; Wald = 7.033; df = 1; *P* = 0.008; Exp(B) = 0.017), and NAA/Cr change (B = -1.424; SE = 0.690; Wald = 4.258; df = 1; *P* = 0.039; Exp(B) = 0.241; Table 3).

**Table 3:** Parameter estimates of the final model

Factor	Relative risk (95% confidence interval)	*P*
1H-MRS metabolite changes 6 months after recovery		
N-acetyl aspartate/creatine	0.238 (0.061-0.921)	0.038
choline/creatine	0.016 (0.001-0.340)	0.008
After dichotomization (only rise or fall monitored)		
N-acetyl aspartate/creatine remains equal or rises	1	
N-acetyl aspartate/creatine decreases	2.015 (1.056-3.844)	0.034
choline/creatine remains equal or rises	1	
choline/creatine decreases	2.063 (1.021-4.167)	0.044

*1H-MRS – proton magnetic resonance imaging.

The model was not significantly improved by dichotomization of variables and the inclusion of composite risk of concomitant Cho/Cr and NAA/Cr direction of changes. Patients who had a decrease in Cho/Cr after remission were at a 2.06 higher risk to experience recurrent episode than patients in whom Cho/Cr ratio remained stable or increased, and those who had NAA/Cr decrease were at a 2.02 higher risk at any given point of time after recovery. The final dichotomized model pointed to Cho/Cr and NAA/Cr decreases as highly predictive variables for the recurrence of a depressive episode.

## Discussion

This study showed that the failure to achieve a rise or at least sustain equal NAA and Cho levels in the DLPFC in the first six months of recovery phase was associated with a doubled risk of the recurrent depressive episode. Identified risks did not display a time-varying effect, so they remained the same during the remaining recovery period.

In line with our previous work, some studies report NAA/Cr increase in the frontal brain regions after SSRI therapy, as well as a Cho increase, but they mostly evaluated patients until entering remission or shortly after it was achieved (16,22,23,31-33). Our research group already reported an increase in Cho/Cr ratio in the DLPFC during antidepressant treatment in early to intermediate responders after 3-6 weeks of SSRI treatment in recurrent depression comorbid to posttraumatic stress disorder (34). Our previous report on the same sample of patients showed that decreased Cho/Cr ratio at the onset of recovery phase predicted depression recurrence in patients on maintenance therapy, and that these patients also exhibited decreased NAA/Cr ratio (28). Our present study focused on all the patients who had depression recurrence whether during maintenance therapy or after the antidepressant withdrawal. By not assuming the knowledge of a future outcome in the present (as in everyday practice), the approach that disregards the duration and outcome of future maintenance therapy offers a more clinically relevant way of looking at recurrence, and suggests the possibility of using NAA and Cho as 1H-MRS markers of longer remission/recovery time before depression recurrence.

The symptoms severity after entering recovery did not change significantly in the observed period. Since a time-varying effect was rejected, it appears that the changes in metabolite levels could not be connected to an imminent worsening of patients’ condition, which we would expect to closely follow the decrease of Cho and NAA metabolites. Observed changes in metabolite levels independent of severity, and their prolonged effect, lead us to the assumption that antidepressant therapy may be related to a temporary improvement (supportive or “symptomatic”) and longer-term brain adaptability or resilience. Sustained NAA and Cho levels at the beginning of the recovery phase may indicate increased brain resilience influenced by antidepressant therapy, but decreased NAA and Cho levels may also indicate only the trait-related temporal effect of therapy in another stratum of patients.

Interestingly, our results may indicate the therapy (antidepressant)-triggered increase in membrane turnover rate in the DLPFC. The observed ratio changes may be the consequence of phosphorylcholine-to-glycerophosphorylcholine mediated synthesis-to-breakdown overbalance, a finding that would be congruent to functional connectivity changes observed in neuroimaging studies of the DLPFC (35,36). Consistent with our previous study (28), we demonstrated that metabolic changes in the brain suggesting an increase in neuronal integrity and membrane turnover continue beyond the acute treatment and remission phases, for at least 6 months after the initiation of maintenance antidepressant therapy. We do not know for how long these changes persist, but this finding certainly warrants the initiation of MRS studies of a considerably longer duration than is our current scientific practice.

Significant metabolite level differences, observed 6 months after the start of recovery, may represent the continuation of a trend in metabolite rise that began at a yet unknown time preceding the recovery phase. If this trend exists, it is impossible to assume whether it is linear or not. Given that significant variability of the time for antidepressant response is well documented, it is likely that metabolites do not start to increase uniformly over time in all patients. This presumed different time-origin of the trend may underlie a wide variability of MRS findings observed in acute and remission phases.

Shorter average time to remission (5.7 weeks) in this group of patients is consistent with lower depression severity at baseline (MADRS of 25.6). Because of the limited range of severity of episodes in our sample, we could not assess the degree to which baseline severity of an episode affected the observed metabolite changes. Also, some previously identified risk factors for recurrence were not included in the analysis (eg, residual symptoms prior to index episode) because verifiable and reliable data on these risk factors could not be gathered for this sample.

It is important to stress the retrospective design as a limitation of our study. Designing and conducting similar but prospective studies with multiple scan points would help with better sample stratification. Also, studies with larger samples are needed, as the present study did not have enough power for any meaningful sensitivity and specificity assessment that would warrant the use in clinical practice. In addition, future studies will need to determine possible effects of non-pharmacological interventions, as increase in Cho was also observed with sleep restriction and transcranial magnetic stimulation (27). All of this would help in discerning the exact nature of metabolite changes as only indirect correlates of different biological processes, or as direct indicators of processes underlying the disease and directing its course and response to treatment.

1H-MRS research of metabolite changes during different phases of illness seems to be indispensable in providing a more detailed insight into the interplay of specific brain metabolites as a putative marker of the longitudinal course of depression. New prospective studies with larger samples, possibly combining different research modalities (MRS, functional connectivity imaging) should make it possible to answer remaining questions and confirm already formed hypotheses, but also to make significant progress in clinical practice. Outcome predictors and the illness course should be identified to help us not only clarify underlying neurobiology and pathophysiological processes, but to formulate meaningful treatment plans with regards to therapy modality and its duration, which would reduce the risk of recurrence and the burden of depression.
